# Evaluation of retina and microvascular changes in the patient with Parkinson’s disease: A systematic review and meta-analysis

**DOI:** 10.3389/fmed.2022.957700

**Published:** 2022-09-15

**Authors:** Yu Deng, Chuanhong Jie, Jianwei Wang, Ziqiang Liu, Yuanyuan Li, Xiaoyu Hou

**Affiliations:** Eye Hospital China Academy of Chinese Medical Sciences, Beijing, China

**Keywords:** Parkinson’s disease, optical coherence tomography, central nervous system, meta-analysis, retina

## Abstract

**Background:**

Parkinson’s disease (PD) is a multifaceted neurodegenerative disease. The optic nerve, as a window into the central nervous system (CNS), is known to be an important part of the CNS and can be detected non-invasively. With the widespread availability of optical coherence tomography (OCT) devices, an increasing number of studies have paid attention to the neuropathological disorders in the retina of PD patients in recent years. However, it is still controversial whether OCT can be used as a complementary tool for PD diagnosis.

**Methods:**

This review is registered with PROSPERO, number CRD42022301258. The Embase, PUBMED, and The Cochrane Library databases were independently retrieved by 2 investigators to identify relevant papers published from 1 January 2017 to 24 January 2022. These studies used OCT or OCTA to evaluate the difference in the retinal nerve fiber layer (RNFL) thickness, ganglion cell layer(GCL) thickness, macula thickness, Cup and disk area superficial retinal capillary plexus (SCP), and deep retinal capillary plexus(DCP). The standard mean difference (SMD) with the 95% confidence interval (CI) was pooled for continuous outcomes.

**Results:**

In total, 26 studies had been enrolled in this meta-analysis with a total number of 2,790 eyes, including 1,343 eyes from the PD group along with 1,447 eyes from the HC group. The results revealed that the RNFL thickness (SMD: −0.53; 95%CI, −0.71∼−0.35; *P* < 0.00001), GCL thickness (SMD: −0.43; 95%CI, −0.66 to −0.19; *P* = 0.0003), macula thickness (SMD: −0.22; 95%CI, −0.22 to −0.11; *P* < 0.0001) were significantly thinner in patients with PD. The SCP (SMD: −0.61; 95%CI, −1.31to −0.10; *P* = 0.02) was significantly lower in PD patients. The DCP (SMD: −0.48; 95%CI, −1.02 to −0.06; *P* = 0.08) is lower in PD patients, but the difference was statistically insignificant.

**Conclusion:**

Retinal nerve fiber layer thickness, GCL thickness, macular thickness, and SVD of PD patients are lower than those of healthy control. OCT and OCTA could detect morphological retinal changes in PD and might be objective and reproducible auxiliary tools to assist clinician diagnosis.

**Systematic review registration:**

[https://www.crd.york.ac.uk/prospero/], identifier [CRD42022301258].

## Introduction

Parkinson’s disease (PD) is often accompanied by severe degeneration of dopaminergic neurons within the substantia nigra pars compacta (1) and pathological changes such as Lewy body formation (2). The clinical symptoms encompass motor and non-motor symptoms such as dyslexia and diplopia (3). Due to the complexity of the pathological process and clinical manifestations of PD, diagnosis of PD and the assessment methods of its progression mainly rely on the clinician’s empirical judgment. However, there is still a lack of diagnostic biological markers (4). Currently, the key challenge is to find a reliable, easily applicable, and well-tolerated diagnostic marker for the diagnosis of PD.

The optic nerve, as part of the CNS (5), has a similar anatomical structure, and physiological characteristics, as well as the same origin of the embryo as the CNS. As the only part of the CNS which can be detected non-invasively, the retina is known as the window into the CNS (6). The structural changes in the retina are the material basis of visual symptoms in PD patients. Results of the post-mortem autopsy showed that PD patients had photoreceptor edema in the retinal plexus layer, loss of retinal ganglion cells (7), decreased retinal dopamine concentration (8), and α –synaptic protein deposition (9). OCT is a non-invasive imaging technique that allows rapid assessment of retinal structural and blood flow changes. Because of its high sensitivity, accuracy, reproducibility, and low cost, it can be used as a potential diagnostic tool for neurodegenerative disorders. However, it is still controversial whether OCT can be used as a biomarker for PD diagnosis. There has been a range of results owing to variations in instruments and research subjects.

Thus, the main purpose of the present systematic review and meta-analysis was to evaluate the difference in the RNFL, macular, GCL, vessel density, and optic disk area between PD patients and health control. The study aimed to provide evidence for the reliability of OCT in the screening and diagnosis of patients with Parkinson’s disease.

## Materials and methods

### Search strategy

This review was registered at PROSPERO (CRD42022301258), and conducted with reference to the Preferred Reporting Items for Systematic Reviews and Meta-Analyses (PRISMA) guidelines (10). EMBASE, PUBMED, the Cochrane library databases were retrieved, while relevant papers were identified from 1 January 2017 to 24 January 2022. Keywords used in the search were “Parkinson’s Disease,” “Lewy Body Parkinson’s Disease,” “Lewy Body Parkinson’s Disease,” Idiopathic Parkinson’s Disease,” “Paralysis Agitans,” “Parkinson’s Disease, Idiopathic,” “Parkinson’s Disease, Idiopathic,” “Parkinson’s Disease,” “Parkinsonism, Primary,” “Idiopathic Parkinson’s Disease,” “Parkinson’s Disease, Lewy Body,” “Primary Parkinsonism,” “Tomography, Optical Coherence,” “Tomography, OCT,” Optical Coherence Tomography” Coherence Tomography, Optical,” “OCT Tomography,”. Using Endnote X20 as a preliminary sieve, references were imported and duplicates removed. Furthermore, references from former related articles were collected to make sure a comprehensive search was conducted.

### Study selection and extraction

Under the guidance of the PICOS statement (participants, interventions, comparisons, outcomes, and study design), two review authors (DY and LZQ) independently determined study eligibility using a standardized inclusion form. Only articles written in English-language were included. Inclusion criteria consisted of: (1) Original article. (2) All patients were clinically diagnosed with Parkinson’s disease and without the medical histories of neither glaucoma, retinal vein obstruction, or other eye diseases. OCT or OCTA was used to observe RNFL thickness as well as other morphological changes. People who matched the subjects for age, and gender were recruited as the healthy control group (HC). (3) Outcome indicators: RNFL thickness, GCL thickness, macular nerve fiber layer thickness, optic disk area. All measurement data are mean ± standard deviation (ME ± SD). (4) The study must be a randomized controlled trial, cross-sectional study, prospective cohort study, or retrospective cohort study. Exclusion criteria were as follows:(1) unclear research question, undefined study object, (2) basic experiments, case series, case reports, meta-analyses, systematic reviews, and commentaries, (3) unable to extract key information from the literature, (4) no control group or incomplete data, (5) did not match the purpose of this article.

Based on the inclusion and exclusion criteria, each piece of literature was independently screened by 2 reviewers(DY and LZQ)and cross-checked. When meeting disagreement, it is necessary to discuss with another reviewer(WJW)to decide whether or not to include studies. The specific screening steps comprised three steps. Firstly, the reviewers would evaluate whether the title and abstract meet the inclusion and exclusion criteria. Secondly, the full text of the remaining articles would be further screened to evaluate whether they meet the eligibility criteria. Finally, two reviewers would check the literature included with each other. When meeting inconsistencies, another reviewer would resolve this problem and determine the inventory of the literature finally included. The extracted information from each study, including the first author, year of publication, study design type, sample size, the mean age of the patients, outcome, and so on, would be carefully and independently extracted by two reviewers.

### Statistical analysis and quality assessment

RevMan version 5.3 software^1^ was used to perform the meta-analysis and draw the risk of bias plots. Instead of mean difference (MD), standardized mean difference (SMD) alone with a 95% confidence interval (95%CI) will be used to assess the continuous outcome, because measurement scales of OCT equipment were consistent between studies. *p* < 0.05 indicated a significant difference, on the other hand, testing for heterogeneity among the included studies was evaluated by the chi-square-based Q test and the I^2^ statistics. When *P* ≥ 0.1 and *I*^2^ ≤ 50%, the possibility of heterogeneity was low and the fixed effect model was adopted. When *P* < 0.1 and *I*^2^ > 50%, the possibility of heterogeneity was high, and the random effect model was adopted. Using egger’s test to judge publication bias when *P* > 0.1 indicated that there was no publication bias. The Newcastle-Ottawa Scale (NOS) was used to evaluate the quality of the included studies for cohort studies.

## Results

**FIGURE 1:**
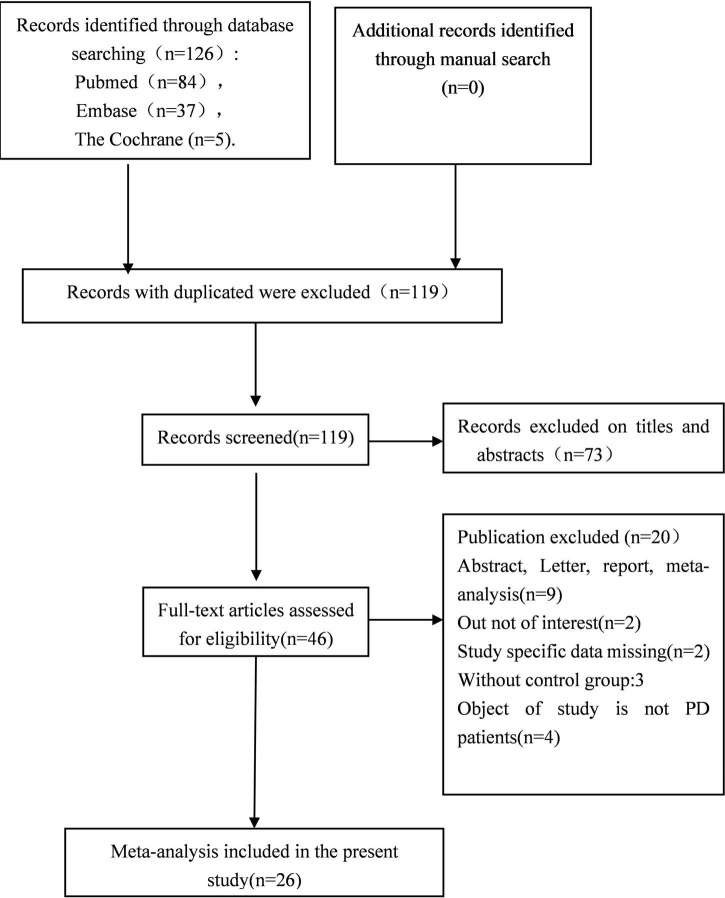
Flow diagram of the study selection process.

The initial search strategy of the four databases retrieved 126 publications in English. A flow diagram of the study selection process is summarized in Figure 1, as well as the basic information of the included studies is shown in Table 1. A total of 119 articles were obtained after removing the duplicates. After screening the titles and abstracts, there are still 46 articles left. Finally, after reviewing the full text 26 articles initially remained: 1 RCT, 3 cohort studies, and 22 cross-section studies. There are a total number of 1,795 participants with 2,790 eyes (PD for 1,343 eyes and HC for 1,447 eyes) extracted from included studies. The sample size ranged from 19 to 137 PD participants, while the average ages of the participants ranged from 52 to 70.72 years old. All studies explicitly described that no statistically significant difference was found in age or gender between the two groups. The average duration of Parkinson’s disease patients ranged from 2.04 to 13.53 years. Out of the 26 studies included in the present meta-analysis, 25 studies explicitly reported the type of the OCT devices, which consisted of Zeiss, Optovue, Heidelberg, Topcon, NIDEK, and VG200. The quality of the included 26 studies was assessed using the Newcastle-Ottawa Scale (NOS) tool, and the quality scores ranged from 7 to 9.

**TABLE 1:** Main characteristics of included studies.

References	Country	Year	Design	Monocular/ Binocular	Group	Sample size (M/F)	Nu of eyes	Age	Mean medical history (Year/ month)	OCT Device	NOS Quality score
Eraslan et al. (11)	Turkey	2016	Cross-section	Binocular	PD	16/9	50	58.64 ± 10.31	53.65 ± 44.13(M)	Zeiss	8
					HC	15/8	46	56.65 ± 9.61	–		
Eraslan et al. (12)	Turkey	2016	Cross-section	Binocular	PD	14/8	44	60.45 ± 9.1	71.41 ± 53.6(M)	Optovue	8
					HC	12/14	50	60.56 ± 9.9	–		
Pilat et al. (13)	Iran	2016	Cross-section	Monocular	PD	19/6	25	60.79 ± 9.24	6.02 ± 1.97(Y)	Optopol	8
					HC	19/6	25	60.58 ± 8.95	–		
Polo et al. (14)	Spain	2016	Cross-section	Monocular	PD	24/14	38	69 (58–74)	13.2 ± 5.77(Y)	Zeiss	8
					HC	24/13	37	68 (60–76)	–		
Satue et al. (15)	Spain	2016	cohort studies	Binocular	PD	17/13	60	69.54 ± 6.6	13.53 ± 6.22(Y)	Heidelberg	8
					HC	17/13	60	68.34 ± 8.45	–		
Ucak et al. (16)	Turkey	2016	Cross-section	Binocular	PD	19/11	58	68.5 ± 7.63	4.87 ± 4.07(Y)	Zeiss	8
					HC	16/14	60	66.23 ± 8.94	–		
Aydin et al. (17)	Turkey	2018	Cross-section	Monocular	PD	17/8	25	70 (50–82)	48(2–192)(M)	Heidelberg	9
					HC	19/10	29	68 (59–78)	–		
Kwapong et al. (18)	China	2018	cohort studies	Monocular	PD	38	49	62.95 ± 7.97	3.84 ± 2.80(Y)	Optovue	8
					HC	28	34	61.18 ± 5.74	–		
Ma et al. (19)	China	2018	Cross-section	Binocular	PD	21/16	74	60.43 ± 8.43	34.95 ± 29.18(M)	Zeiss	7
					HC	23/19	84	57.31 ± 9.54	–		
Matlach et al. (20)	Germany	2018	Cross-section	Binocular	PD	21/9	46	64.1 ± 8.3	9.8 ± 6.9(Y)	Zeiss	8
					HC	18/22	40	64.1 ± 8.2	–		
Moschos et al. (21)	Greece	2018	Cross-section	Monocular	PD	17/14	31	67.8 ± 3.9	–	Heidelberg	9
					HC	14/11	25	68.0 ± 4.1	–		
Satue et al. (22)	Spain	2018	Cross-section	Monocular	PD	35/15	50	70.72 ± 6.20	13.53 ± 6.22(Y)	Topcon	8
					HC	35/19	54	69.57 ± 8.13	–		
Unlu et al. (23)	Turkey	2018	Cross-section	Binocular	PD	15/13	56	59.06 ± 9.41	10.80 ± 6.15(Y)	Heidelberg	8
					HC	15/15	60	60.22 ± 13.41	–		
Visser et al. (24)	Netherlands	2018	Cross-section	Binocular	PD	15/5	39	65 (54–70)	8.0 (4, 15)(Y)	Heidelberg	8
					HC	9/11	39	52 (63–75)	–		
Elkhatib et al. (25)	Egypt	2019	cohort studies	Binocular	PD	10/10	40	63.2 ± 5.50	6.53 ± 3.07(Y)	NIDEK	9
					HC	11/9	40	62.4 ± 6.96	–		
Gulmez Sevim et al. (26)	Turkey	2019	Cross-section	Monocular	PD	20/20	41	59.64 ± 9.94	4(Y)	Heidelberg	7
					HC	19/16	35	59.44 ± 7.59	–		
Hasanov et al. (27)	Turkey	2019	RCT	Binocular	PD	19	38	54.39 ± 5.71	47.21 ± 41.15(M)	Topcon	7
					HC	19	38	55.53 ± 6.48	–		
Murueta-Goyena et al. (28)	Spain	2019	cross section	Binocular	PD	41/22	126	61.91 ± 8.56	8.81 ± 5.15(Y)	Heidelberg	8
					HC	16/18	68	59.79 ± 6.22	–		
Shafiei et al. (29)	Iran	2019	Cross-section	Monocular	PD	18/5	23	61.30 ± 11.57	–	–	8
					HC	18/5	23	61.22 ± 11.39	–		
Sung et al. (30)	Korea	2019	Cross-section	Monocular	PD	25/49	74	65.30 ± 8.38	–	Zeiss	9
					HC	18/35	53	64.68 ± 6.64	–		
Rascuna et al. (31)	Italy	2020	Cross-section	Binocular	PD	12/9	41	59.3 ± 7.0	27.4 ± 14.3(M)	Zeiss	8
					HC	9/8	33	/			
Shi et al. (32)	China	2020	Cross-section	Binocular	PD	12/13	50	61.9 ± 7.6	3.7 ± 2.4(Y)	Optovue	9
					HC	12/13	50	59.0 ± 5.8	–		
Tugcu et al. (33)	Turkey	2020	Cross-section	Binocular	PD	12/9	42	62.48 ± 9.76	5.02 ± 3.25(Y)	Heidelberg	9
					HC	12/10	43	62.41 ± 6.99	–		
Robbins et al. (34)	Singapore	2021	Cross-section	Binocular	PD	39/30	124	71.7 ± 7.0	–	Zeiss	8
					HC	77/60	248	70.9 ± 6.7	–		
Zhang et al. (35)	China	2021	Cross-section	Binocular	PD	45	75	55.92 ± 7.53	2.04 ± 1.23(Y)	VG200	9
					HC	75	150	54.68 ± 6.66			
Zhou et al. (36)	China	2021	Cross-section	Monocular	PD	18/6	24	65.88 ± 6.50	5.3 ± 4.2(Y)	Zeiss	8
					HC	11/12	23	63.43 ± 7.11			

### Retinal nerve fiber layer thickness

**FIGURE 2:**
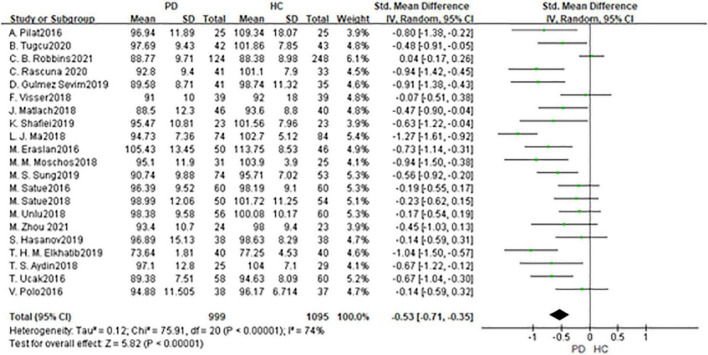
Forest plot of the retinal nerve fiber layer (RNFL) thickness between PD group and HC group. PD, Parkinson’s disease; HC, health control.

In total, 21 studies had been enrolled in this meta-analysis with a total number of 2,094 eyes, including 999 eyes from the PD group, while 1,095 eyes from the HC group (Figure 2). First, the heterogeneity test was conducted. The result shows that there was high heterogeneity in the study (*I*^2^ = 74%). After removing articles one by one, the *heterogeneity* remained substantial, therefore a random-effects model was used for the analysis. The results indicated that RNFL thickness in the PD group was significantly thinner than in the HC group (SMD: −0.53; 95%CI, −0.71∼−0.35; *P* < 0.00001). Additionally, the result is consistent with that of the most included studies (11, 13, 16, 17, 19–21, 25–27, 29–31, 33). However, seven studies showed no statistically significant difference in RNFL between PD and HC groups (14, 15, 22–24, 34, 36). Furthermore, the meta-analysis of RNFL thickness in superior, inferior, nasal, and temporal quadrants showed that RNFL thickness in the PD group of all quadrants, especially in superior and inferior quadrants, was thinner than HC group [(RNFL-S: SMD: 0.53; 95%CI, 0.81–0.26; *P* = 0.0001);(RNFL-I: SMD: 0.53; 95%CI, 0.80–0.26; *P* = 0.0001); (RNFL-N: SMD: 0.22; 95%CI, 0.37–0.07; *P* = 0.003);(RNFL-T: SMD:−0.25; 95%CI,−0.45 to −0.06; *P* = 0.009)] (Table 2). The Egger test was used to evaluate the publication bias, and the result showed P = 0.2977, indicating no obvious publication bias.

**TABLE 2:** The results of the meta-analysis of the RNFL thickness in different quadrants.

	No of studies	N (PD/HC)	*I*^2^(%)	*P*	Model	SMD&95%CI	Eggers test
RNFL-S	14	619/594	81%	=0.0001	Random(IV)	−0.53 [−0.81, −0.26]	0.0590
RNFL-I	14	578/561	81%	=0.0001	Random(IV)	−0.53 [−0.80, −0.26]	0.0137
RNFL-N	16	719/710	51%	=0.003	Random(IV)	−0.22[−0.37, −0.07)]	0.1811
RNFL-T	15	694/681	69%	=0.009	Random(IV)	−0.25[−0.45, −0.06)]	0.2114

### Ganglion cell layer thickness

**FIGURE 3:**
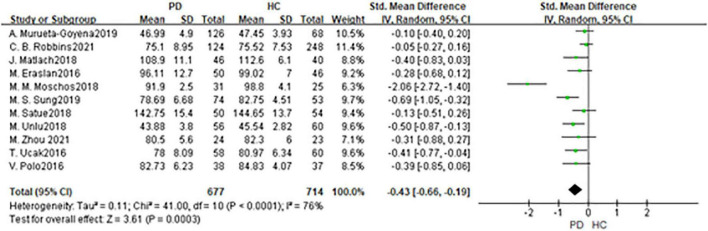
Forest plot of the GCL thickness between PD group and HC group. PD, Parkinson’s disease; HC, health control; GCL, Ganglion Cell Layer.

In total, 11 studies had been enrolled in this meta-analysis with a total number of 1,391 eyes, including 677 eyes from the PD group, while 714 eyes from the HC group (Figure 3). First, the heterogeneity test was conducted. The result shows that there was high heterogeneity in the studies (*I*^2^ = 76%). After removing articles one by one, the *heterogeneity* remained substantial, therefor a random-effects model was used for the analysis. The results indicated that GCL thickness in the PD group was significantly thinner than in the HC group (SMD: −0.43; 95%CI, −0.66 to −0.19; *P* = 0.0003). Additionally, the result is consistent with the 4 included studies (16, 21, 23, 30). Nevertheless, seven studies showed no statistically significant difference in GCL between PD and HC groups (11, 14, 20, 22, 28, 34, 36). The Egger test was used to evaluate the publication bias, and the result showed *P* = 0.4285, indicating no obvious publication bias.

### Cup and disk area

**FIGURE 4:**
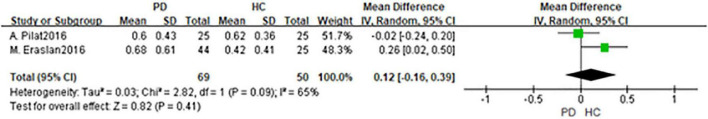
Forest plot of the disk area between PD group and HC group. PD, Parkinson’s disease; HC, health control.

**FIGURE 5:**
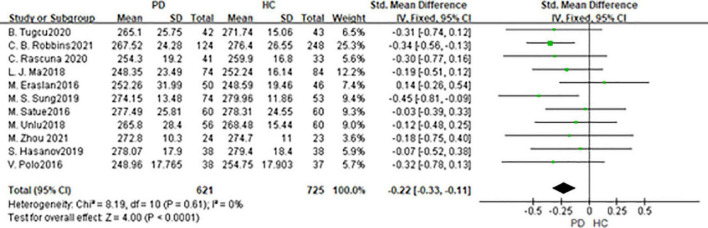
Forest plot of the cup area between PD group and HC group. PD, Parkinson’s disease; HC, health control.

In total, 2 studies had been enrolled in this meta-analysis with a total number of 144 eyes, including 69 eyes from the PD group, and 75 eyes from the HC group (Figures 4, 5). First, the heterogeneity test was conducted. The result shows that there was heterogeneity in the study (*I*^2^ = 49%), so the random-effects model was used for the analysis. The results indicated that there was no statistically significant difference in a cup and disk area among the PD group and the HC group (*P* = 0.45; *P* = 0.39).

### Macula thickness

**FIGURE 6:**
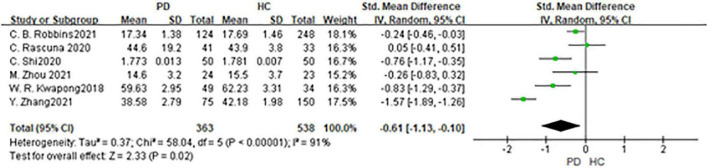
Forest plot of the macula thickness between PD group and HC group. PD, Parkinson’s disease; HC, health control.

In total, 11 studies had been enrolled in this meta-analysis with a total number of 1,346 eyes, including 621 eyes from the PD group, while 725 eyes from the HC group (Figure 6). First, the heterogeneity test was conducted. The result shows that there was no heterogeneity in the study (*I*^2^ = 0%), so the fixed-effects model was used for the analysis. The results indicated that macula thickness in the PD group was significantly reduced compared with the HC group (SMD: −0.22; 95%CI, −0.22 to −0.11; *P* < 0.0001). Nevertheless, there were only two studies consistent with our finds (30, 34). No statistically significant difference was found between the PD and HC groups in the other nine studies (11, 14, 15, 19, 27, 31, 33, 36). The Egger test was used to evaluate the publication bias, and the result showed *P* = 0.0688, indicating the existence of publication bias.

### Vessel density in superficial retinal capillary plexus

**FIGURE 7:**
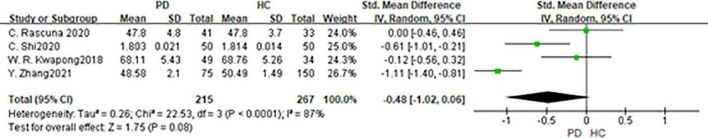
Forest plot of the SCP between PD group and HC group. PD, Parkinson’s disease; HC, health control.

Six studies had been enrolled in this meta-analysis with a total number of 901 eyes, including 363 eyes from the PD group, and 538 eyes from the HC group (Figure 7). First, the heterogeneity test was conducted. The result shows that there was high heterogeneity in the study (*I*^2^ = 91%). After removing articles one by one, the *heterogeneity* remained substantial, therefore the random-effects model was used for the analysis. The results indicated that SCP in the PD group was significantly lower than in the HC group (SMD: −0.61; 95%CI, −1.31 to −0.10; *P* = 0.02). Furthermore, a meta-analysis of SCP in superior, inferior, nasal, and temporal quadrants showed that SCP in the PD group of all quadrants, especially in nasal quadrants, was significantly lower than in the HC group [(SCP-S: SMD: −0.45; 95%CI, −0.75 to −0.15; *P* = 0.003);(SCP-I: SMD:−0.59; 95%CI, −1.17 to −0.02; *P* < 0.0001); (SCP-N: SMD: −0.92; 95%CI, −1.34 to −0.50; *P* < 0.0001);(SCP-T: SMD:−0.59; 95%CI,−0.79 to −0.40; *P* < 0.0001) (Table 3). The Egger test was used to evaluate the publication bias, and the result showed *P* = 0.1397, indicating no obvious publication bias.

**TABLE 3:** The results of the meta-analysis of the SCP in different quadrants.

	No of studies	N (PD/HC)	*I*^2^(%)	*P*	Model	SMD&95%CI	Eggers test
SCP-S	4	239/290	62%	=0.003	Random (IV)	−0.45 [−0.75, −0.15]	0.1654
SCP-I	4	239/290	89%	<0.00001	Random (IV)	−0.59 [−1.17, −0.02]	0.2848
SCP-N	4	198/257	74%	<0.00001	Random (IV)	−0.92 [−1.34, −0.50]	0.1545
SCP-T	4	198/257	0%	<0.00001	Fixed (IV)	−0.59 [−0.79, −0.40]	0.0735

### Vessel density in deep retinal capillary plexus

**FIGURE 8:**
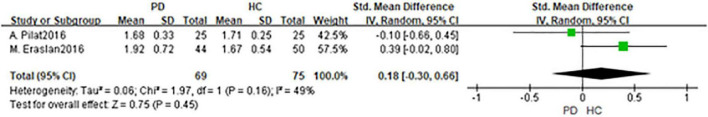
Forest plot of the DCP between PD group and HC group. PD, Parkinson’s disease; HC, health control.

Three studies had been enrolled in this meta-analysis with a total number of 482 eyes, including 215 eyes from the PD group, and 267 eyes from the HC group (Figure 8). First, the heterogeneity test was conducted. The result shows that there was heterogeneity in the study (*I*^2^ = 87%). After removing articles one by one, the *heterogeneity* remained substantial, so the random-effects model was used for the analysis. The results indicated that DCP in the PD group was no statistical difference from that in the HC group (SMD: −0.48; 95%CI, −1.02 to −0.06; *P* = 0.08). Furthermore, the meta-analysis of DCP in superior, inferior, nasal, and temporal quadrants showed that DCP of superior, inferior, and nasal quadrants in the PD group were significantly reduced compared with HC group [(DCP-S: SMD: −0.91; 95%CI, −1.82 to 0.00; *P* = 0.05);(DCP-I: SMD: −0.75; 95%CI, −1.48 to −0.02; *P* = 0.04); (DCP-N: SMD: −0.45; 95%CI, −0.88 to −0.01; *P* = 0.04)] (Table 4). However, there was no statistically significant difference between the PD group and the HC group in the temporal quadrant (DCP-T: SMD:−0.59; 95%CI,−1.28 to 0.10; *P* = 0.09) (Table 3). The Egger test was used to evaluate the publication bias, and the result showed *P* = 0.3848, indicating no obvious publication bias.

**TABLE 4:** The results of the meta-analysis of the DCP in different quadrants.

	No of studies	N (PD/HC)	*I*^2^(%)	*P*	Model	SMD&95%CI	Eggers test
DCP-S	3	215/267	95%	0.05	Random (IV)	−0.91 [−1.82, 0.00]	0.3738
DCP-I	3	215/267	91%	0.04	Random (IV)	−0.75 [−1.48, −0.02]	0.4211
DCP-N	3	174/234	75%	0.04	Random (IV)	−0.45 [−0.88, −0.01]	0.3848
DCP-T	3	174/234	90%	0.09	Random (IV)	−0.59 [−1.28, 0.10]	0.4795

## Discussion

In the present meta-analysis, we directly assessed OCT parameters such as the RNFL thickness, GCL thickness, macula thickness, etc., to evaluate neuropathological changes in PD patients. PD patients are often accompanied by visual symptoms such as visual illusions and minor hallucinations. A post-mortem study revealed that retinal dopamine content was reduced in PD patients (37), and the GCL, inner plexiform layer (IPL) as well as RNFL thinning were also found in PD patients (38). Decreased dopamine secretion and degeneration of retinal dopaminergic neurons are directly related to visual impairment and retinal nerve changes in PD patients (39). The neuropathological disorders in the retina of PD patients have gradually attracted more attention in recent years (40). RNFL thickness thinning in PD patients was first reported in 2004 by Martin et al. (41), they used OCT to demonstrate neuropathological changes in PD patients. Yet, other publications come to varying conclusions. Aaker et al. (42) reported no significant difference in RNFL thickness among the groups. Robbins et al. (34) reported that RNFL thickness in PD patients was a little bit higher than HC group but there was still no statistical difference. Nevertheless, Satue et al. (43) used OCT to evaluate retinal changes in eyes of PD patients and the results showed that PD led to RNFL, GCL, and macula thinning. Because of the discrepancy between former studies, whether OCT scan was able to identify retinal changes in PD patients and classify patients reliably into the patient group is still controversial.

The present study found that RNFL, GCL, and macula thickness are thinning in PD patients, moreover, OCT can reliably classify the PD patients according to retinal changes. Structural and functional retinal changes, such as thinning in RNFL thickness, have been described in a variety of neurodegenerative diseases like multiple sclerosis and Alzheimer’s disease (44), suggesting that retinal degeneration may occur simultaneously with central neurodegenerative changes. Therefore retina has been suggested to be the window to the neuropathological changes of the central nervous system (45). Garcia et al. (46) found that the thickness of RNFL in PD patients was negatively correlated with the severity of PD. Powell et al. (47) found that RNFL gradually became thinner by using an OCT device, and RNFL thickness was negatively correlated with the severity and duration of PD. Thus, using OCT to monitor dynamic retinal changes of PD patients is of Therefore, monitoring RNFL thickness in PD patients is of great importance in the early diagnosis as well as monitoring the development of the PD patients. Since only 1/10 of the retinal nerves are dopaminergic neurons (48), the loss of dopaminergic neurons has little influence on the thickness of RNFL. That may be the reason for the absence of statistical significance in RNFL changes in some studies. In this study, it was found that the thinning of RNFL was most obvious in the superior and inferior quadrants of PD patients, which may be related to the gradual degeneration of dopaminergic neurons in the retinal ganglion cells and amacrine cells, and eventually leading to the apoptosis of retinal ganglion cells (49). Finally led to the gradual atrophy of the optic nerve. The macula is the most sensitive part of the retina, where more than 50% of the retinal ganglion cells are concentrated. The changes in contrast sensitivity and color vision of PD patients are related to the thinning of GCL in the macula (50). Garcia et al. (46) reported that the GCL thickness of PD patients was negatively correlated with PD severity, while Polo et al. (14) found that GCL thickness was moderately correlated with color vision and contrast sensitivity. The clinical manifestations of PD are complex and varied, meanwhile different patients often suffer from different combinations of motor and non-motor symptoms. At present, there is a lack of reliable and easily detected biological markers (51). On the one hand for complicated PD patients who are unable to undergo lumber puncture pathological diagnosis and cannot be diagnosed and differentiated, OCT can be an important and useful adjunct for early diagnosis. On the other hand, for patients definitively diagnosed with PD, OCT is also an important adjunct to monitoring disease progression. In general, retinal changes play an important role in the progress of PD, on the other hand, OCT scan is a useful adjunct to differentiate people with PD from healthy controls.

As a promising non-invasive technique that can be used for imaging the microvasculature of the retina, optical coherence tomography angiography (OCTA) enables doctors to get a quantitative and rapid characterization of the retinal capillary in different layers (52). OCTA can provide not only structural and functional images of retinal vasculatures without using contrast agents but also better visibility (53). OCTA can detect retinal microvascular abnormalities of superficial and deep layers in patients who have no detectable clinical retinopathy (54). Former studies have shown that the retinal microvascular density decreased in PD patients (18), meanwhile, OCTA can be used as one of the biological indicators for the early diagnosis of PD (55). The SCP and DCP can be measured by OCTA scanning. Robbins et al. (34) found that vessel density (VD) decreased in PD patients, however, no structure changes such as tinning in RNFL were found in their study. Kwapong et al. (18) found that the SCP of PD patients decreased significantly, however, there was no statistically significant difference in the DCP. Rascuna et al. (31) found there was no significant difference in the SCP among the groups.

There is limited literature on retinal microvascular disorders in patients with PD, by OCTA scanning. As far as we know, this paper is the first systematic review of retinal microvascular density in patients with PD by OCTA scanning. The present study found that the SCP in PD patients was lower than that of the HC group. The result is in good agreement with former studies (18, 32, 34, 36). Additionally, two studies are inconsistent with our finds, Zhou et al. (36) found that the SVD of PD patients was a little bit lower than HC group, but the difference was not statistically significant, Rascuna et al. (31) found that the SVD was slightly higher in PD group, however, the difference was not statistically significant. A further meta-analysis of VD in different quadrants showed that SCP in PD patients in all quadrants was lower than that in the HC group, with the greatest reduction in nasal-SCP, with statistical significance (*P* < 0.05). Inflammation is the key pathogenesis and potential therapeutic target of PD (56); α-synuclein (α-syn), which also called Lewy bodies or Lewy neurites, is the core pathological feature of PD (57). The decreased VD and FAZ perimeter in PD patients are closely related to retinal neuroinflammation and gliosis (34). Ortuno et al. (58) reported retinal α –syn deposition around the retinal artery in mice with PD. Decreased retinal thickness and VD can also be observed in other degenerative neuropathies like multiple sclerosis (59), and the retinal microvascular degenerative pathologies, characterized by abnormal changes in VD, reflect processes of retinal degeneration (34). Compared with RNFL and other indicators, SCP may be an earlier and more sensitive indicator for PD patients (35). OCT in combination with OCTA can improve the diagnostic accuracy of PD (36). Meta-analysis of the DCP indicated that there was no significant difference between PD and HC groups. Further analysis of DCP in each quadrant showed that inferior and nasal DCP were lower than HC group, and the difference was statistically significant (*P* < 0.05), while superior and temporal DCP had no significant difference. DVD is greatly affected by projection artifacts and different OCTA devices are probable to have different OCTA algorithms (60), this could be the reason why the changes in the DCP in PD patients were not obvious.

Accounting for decreased RNFL thickness with advancing age, the average RNFL thickness decreased at a range of 0.4 μm per year (61). There was a large difference in the age between the included studies, for example, the mean age of the study by Eraslan et al. (11) is 10 years older than that reported by Polo et al. (14), while the mean duration was 7 years longer in the study by Eraslan et al. (11). The right and left sides of the brain are asymmetrically affected in PD patients (62), Shriert et al. (63) and Cubo et al. (64) found that there was intraocular asymmetry in macular retina thickness.10 studies of the included 26 studies randomly selected one eye from each patient for the analysis, on the contrary, 16 studies selected two eyes for the analysis. The difference in the selection of eyes might be an important reason for the heterogeneity. It is worth noting that a single retinal parameter may not be discriminative enough to serve as an independent biomarker with a predefined cutoff value to define disease presence or absence. However, these OCT and OCTA image findings may enhance clinician confidence in the diagnosis of PD when combined with clinical history and other existing tests. There is a need for future long-term studies that characterize the natural history of microvascular and structural changes in retinal tissue across the clinical spectrum of Parkinson’s disease. The results of such studies may provide insight into whether they can be used to assess the onset or rapid progression of PD.

However, the following limitations still exist in this study. First, retinal thickness is correlated with retinal dopamine concentrations. It is unclear whether dopaminergic medications have any effect on measurements obtained from OCT or OCTA. As an effective treatment for PD patients, dopaminergic medications are the drug of choice to relieve motor and non-motor symptoms of PD patients. Additionally, a majority of the included studies didn’t illustrate whether PD patients take dopaminergic medications, which may lead to bias in the results and consequently affect the accuracy of it. This may be the reason for the differences in the results of different studies. Second, different OCT devices of different manufacturers have different scanning strategies (65) which led to different scanning artifacts (66). Inter-device differences may cause bias in the present meat-analysis. Finally, refractive media opacity may affect the result of VD measured by OCTA (67), most of the included studies don’t illustrate whether all participants have clear refractive media. Limited by the existing devices as well as algorithm, the VD of DCP can be disturbed by SCP (68). Further studies must consider these limits (69).

## Conclusion

Retinal nerve fiber layer thickness, GCL thickness, macular thickness, and SVD of PD patients are lower than those of healthy control. Serving as supplementary diagnostic tools, OCT and OCTA could detect early morphological retinal changes in PD and might be objective and reproducible auxiliary tools to assist clinician diagnosis. In the future, OCT and OCTA can be used to judge the progression of PD.

## Data availability statement

The original contributions presented in this study are included in the article/supplementary material, further inquiries can be directed to the corresponding author.

## Author contributions

CJ contributed to the conception and design of the study. JW, ZL, XH, and YL retrieved the articles and organized the database. YD performed the statistical analysis and wrote sections of the manuscript. All authors contributed to manuscript revision, read, and approved the submitted version.

## Footnotes

1
www.cochrane.org
